# Interference of Cadmium with the Early Development of *Artemia salina*

**DOI:** 10.3390/toxics14070609

**Published:** 2026-07-12

**Authors:** Chiara Maria Motta, Bice Avallone, Chiara Fogliano, Raffaele Panzuto, Elisabetta Piva, Sara Pacchini, Paola Venditti, Gianluca Fasciolo, Patrizia Cretì, Salvatore De Bonis, Simona Di Marino, Rosa Carotenuto

**Affiliations:** 1 Department of Biology, University of Naples Federico II, 80126 Naples, Italy; mottacm@unina.it (C.M.M.); bice.avallone@unina.it (B.A.); paola.venditti@unina.it (P.V.); gianluca.fasciolo@unina.it (G.F.); simona.dimarino@unina.it (S.D.M.); 2 Conservation of Marine Animals and Public Engagement, Stazione Zoologica Anton Dohrn, 80122 Naples, Italy; raffaele.panzuto@szn.it; 3 Department of Biology, University of Padova, 35122 Padova, Italy; elisabetta.piva.2@studenti.unipd.it (E.P.); sara.pacchini@studenti.unipd.it (S.P.); 4 Department of Biological and Environmental Sciences and Technology, University of Salento, 73100 Lecce, Italy; patrizia.creti@unisalento.it; 5 Regional Agency for Environmental Protection of Lazio, 00187 Rome, Italy; salvatore.debonis@arpalazio.it

**Keywords:** oxidative stress, cadmium uptake, brush border, yolk resorption, eye damage

## Abstract

Cadmium is a common contaminant of both saline and freshwater environments. It has no biological function but readily penetrates tissues, causing significant oxidative stress and consequent damage throughout the organism. In this work, we examined the effects of this metal on the nauplii of *Artemia salina*, a model for testing toxicity in larval zooplanktonic species, and to evaluate the impact of contaminants on natural populations. An environmentally realistic concentration (15 µg/L) and two higher concentrations (150 and 1500 µg/L) were used. The presence of metals in culture media and nauplii was verified by atomic absorption spectroscopy, while oxidative stress levels were assessed by measuring hydroperoxides, carbonyl groups, ROS, and total antioxidant capacity. Mortality and growth rates were the conventional toxicity endpoints analysed. In parallel, morphological alterations were assessed in toto and in section. Attention was directed to the gut, the primary site of metal uptake and reabsorption, and to effects on protein patterns. The results demonstrated significant dose-independent Cd uptake by the nauplii and dose-dependent oxidative stress. Consequent alterations in brush border organisation and yolk and lipid resorption were assessed. Modest teratogenic effects were detected in naupliar eye pigmentation and paired-eye organisation. Oxidative stress and the marked changes in protein pattern justify the alterations observed, while the upregulation of the *hsp26* and *hsp60* genes indicates naupliar attempts to mitigate damage. In conclusion, this study confirms the effects of Cd toxicity on the development of *Artemia salina* nauplii and demonstrates interference with reserve resorption. This evidence indicates that this heavy metal can have a profound impact on zooplanktonic communities, which are essential to the stability of the entire aquatic food web.

## 1. Introduction

Cadmium is a contaminant in freshwater and marine environments and a cause of worldwide concern [1]. In surface waters, it reaches concentrations ranging from 13 ng/L [2] to about 700 µg/L in severely polluted areas near mines [3]. Its origin, concentration and effects on biota are the object of numerous reviews [4,5,6].

Cadmium has no biological function; however, it is readily taken up and bioaccumulated [7], especially in detoxifying organs [8]. To a lesser extent, it also accumulates in all tissues, including the gills, gut, and heart [9,10]. Once in the cell, the metal induces oxidative stress [11] and damages DNA [12], eventually dysregulating gene expression [13].

Cadmium is also an endocrine disruptor [14]. It accumulates in the gonads [15] and interferes with both oogenesis [16] and spermatogenesis [17]. Sperm vitality, fertilisation [18], and overall fecundity are consequently impaired.

In embryos, cadmium dysregulates gene expression [13], leading to teratogenic effects. These range from altered body axes [19] to impaired skeletal mineralisation [20], and from the brain [21] to retinal deformities [22] and gut malrotation [23]. Direct interference with larval survival [24] is likewise reported.

Effects on crustacean larvae have received comparatively little attention, despite the importance of this group of invertebrates within the microzooplankton. Nauplii feed on and control phyto- and nano-zooplanktonic species. In addition, they are preyed upon by larger predators, thereby representing a fundamental link in the food web [25]. Nauplii are small, filter-feeding animals that are highly vulnerable to metal contamination. Cd is absorbed not only through food but also via the gills and skin, which provide a large surface area for contact with contaminated water [26]. Studies on metal uptake mainly report LC50 values or describe effects on mortality, immobilisation, and swimming speed [27,28]. Therefore, this study aimed to identify novel targets of cadmium toxicity in early naupliar (larval) development. The study was conducted on the lecithotrophic species *Artemia salina*, a valuable crustacean model organism. Nauplii are economical, easy to handle, and widely used in environmental risk assessment [29]. Three nominal concentrations of Cd^2+^ were tested: 15, 150, and 1500 µg/L. The first two are close to the environmental concentrations recorded at polluted sites [30] and within the range in which nauplii balance accumulation with elimination rates [31]. The latter falls within the concentration range that promotes tissue accumulation [31]. All three concentrations are significantly lower than the LC10 for adults (58 mg/L) [28].

The effects of exposure were assessed using a multidisciplinary approach. To verify Cd toxicity in our model, conventional endpoints, such as hatching rate, mortality, and growth [32], were examined first. Parallel investigations were conducted to assess the occurrence of teratogenic alterations in toto and in sections. Particular attention was directed to resorption of yolk reserve, abundant in this species [33], and primary food sources during the first hours of post-hatching life [34]. Previous evidence indicated that changes in yolk carbohydrate composition and delayed resorption occur after exposure to a benzodiazepine [35] and polystyrene microbeads [32]. Other targets were the eyes and the gut epithelium, the latter of which is responsible for the uptake of approximately 30% of the cadmium accumulated in these larvae [36].

The results were correlated with cadmium concentrations, determined in culture water and nauplii by atomic absorption spectroscopy. Naupliar oxidative stress (ROS, lipid and protein peroxidation, and total antioxidant capacity) was also determined, along with changes in protein pattern. The expression of two heat shock proteins, *hsp26* and *hsp60*, was also assessed, as they control stress-induced protein misfolding following exposure to environmental toxicants [29].

## 2. Materials and Methods

### 2.1. Maintenance and Treatment of Artemia salina

A total of 250 mg of *Artemia salina* cysts, purchased from a commercial source (routinely used in our laboratory, with a guaranteed hatching rate > 92%), were hydrated in 500 mL of 35‰ artificial seawater prepared with 35 g/L Instant Ocean Salt [37]. The cultures were kept in 750 mL glass tanks at 21 ± 1 °C and pH 7.4, with a 12 h dark/12 h light photoperiod (light intensity about 30 lumens/L). Hatching occurred 30 ± 1.5 h after hydration started. The nauplii were fed twice daily with a commercial preparation specifically designed for *Artemia salina* (a product routinely used in our laboratory that consists of unicellular algae) and brewer’s yeast [38]. The mortality rate in control samples never exceeded 10% at 2 days post-hatching (dph) [37]. These cultures were used to evaluate naupliar mortality, growth and phenotypic and biomolecular alterations (see Section 2.4, Section 2.5, Section 2.6, Section 2.7, Section 2.8, Section 2.9 and Section 2.10).

Three sublethal concentrations were tested. A stock solution of 3 mg/L CdCl_2_ (Carlo Erba, Cornaredo, Italy, purity ≥ 98%) and two dilutions (10- and 100-fold) were prepared with seawater and used for culture. The nominal Cd^2+^ concentrations of the three working solutions were ~1500, ~150 and ~15 μg/L, respectively (Visual MINTEQ, version 3.1). Exposure was static (no water changes) [32] and lasted from hydration to five days post-hatching.

### 2.2. Evaluation of Cadmium Concentration

To determine naupliar uptake, cadmium concentrations were measured in culture seawater and in nauplii at 5 days post-hatching (dph), the end of the experiment. Nauplii (n = 4 replicates/treatment) were first collected from cultures using a sieve and then digested with concentrated nitric acid (65%, Ultrapure, Fluka). The mixture was heated for 15 min at 70 °C, cooled, and centrifuged for 5 min at 12,000× *g*, at room temperature. Metal contents were determined by Graphite furnace atomic absorption spectrophotometry Perkin Elmer 5100 (PerkinElmer Inc., Shelton, CT, USA) equipped with Zeeman graphite furnace). Parallel measures were conducted on seawater and cysts (˂1 µg/L and 0.01 µg/gr, respectively). Ultrapure water and standard stock solution of the metals (1 mg/mL) were obtained from a commercial source (Agilent Technologies, Santa Clara, CA, USA; purity 99.9%). Working standards in 0.2% *v*/*v* HNO_3_ were prepared by diluting known aliquots of the stock solution to the appropriate volume. The standard addition curve was used to determine the Cd detection limit in different samples. It was based on the usual definition of analyte concentration, yielding a signal equivalent to 3 times the standard deviation of the reagent blank (n = 5). The estimated detection limit (LOD) was 1–5 ng/g.

### 2.3. Hatching Test

An average of 100 ± 17 cysts were placed in 3 mL of seawater (control) or in seawater containing cadmium at one of three concentrations to be tested. Four replicates were prepared in a 24-well plate (Corning^®^, New York, NY, USA; polystyrene). Cultures, conducted at 21 ± 1 °C and pH 7.4, were maintained in the dark until the first control nauplius emerged from the cyst. After 12 h, nauplii and cysts were harvested. Early sampling enabled the interception of embryos with accelerated development. Nauplii and cysts were immediately fixed in 75% ethanol and counted under a stereo microscope (Zeiss, Oberkochen, DE, Germany; OPMI 1-F). The ratio of nauplii to cysts × 100 was used to calculate the hatching percentage. Six separate experiments were conducted, and in each experiment, data were transformed into percentages relative to the respective control to reduce bias introduced by different cyst batches. Values were then pooled and expressed as means ± SD (n > 2000 cysts/treatment).

### 2.4. Mortality

Every 12 h after hatching, for five days, five aliquots of 1 mL each were collected from the 500 mL culture (Section 2.1). They were immediately observed to determine the number of vital and dead nauplii (immobile for at least 15 s). The ratio of vital nauplii to dead nauplii × 100 was used to calculate the mortality percentage. Six replicate cultures were conducted, and in each test, treatment data were expressed as a percentage of the control to reduce bias introduced by differences among cyst batches. Values were pooled and expressed as means ± SD (n > 2000 nauplii/treatment).

### 2.5. Growth Test

Daily, from hatching to 5 dph, three random 10 mL aliquots were taken from the 500 mL cultures (Section 2.1). The nauplii were fixed in 75% ethanol and observed under a microscope to determine their developmental stage and number (6 tests; n > 3000 nauplii/treatment). The staging was according to [35]. The ratio of nauplii at different stages to the total number of nauplii examined, expressed as a percentage, indicated the population composition.

### 2.6. Phenotypic Analyses

Fixed nauplii were observed under a light microscope to verify proper development of the body axes, thoracopods, and naupliar and paired eyes, proven targets of toxicity in *Artemia salina* [35]. Determinations were conducted on digital photos of nauplii at specific stages of development (n = 100 per treatment per replicate experiment). For naupliar eyes, pigment intensity was also assessed. Naupliar heads (n = 100 per treatment) were photographed at the L3/L4 stage, and absorbance was measured from the high-resolution images using ImageJ (version 1.54k, accessed Nov 2025). Body density was measured to assess reserve resorption [32]. Nauplii at the L3/L4 stage (n = 50 per treatment) were photographed, and absorbance was recorded from the high-resolution images using ImageJ (version 1.54k, accessed Nov 2025). Selected measurement areas included both the body wall and the gut margin.

### 2.7. Oxidative Stress in 5-dph Nauplii

Lipid peroxidation was evaluated by measuring lipid hydroperoxide (HPs). Aliquots of homogenates containing 10 µg of protein were diluted in 0.1 M PBS, pH 7.4 [39]. The decrease in NADPH absorbance was monitored at 340 nm, in the presence of GSH. Lipid hydroperoxide levels were expressed as nmol NADPH oxidised per minute per milligram of protein. Protein damage was quantified by measuring the reaction of protein-bound carbonyls (CO) with 2,4-dinitrophenylhydrazine (DNPH) at 450 nm. Briefly, homogenates were diluted in PBS (pH 7.4) containing 0.2% digitonin and an antiprotease cocktail, incubated for 10 min, and centrifuged at 600 *g* at 4 °C. The supernatant was treated with 1% streptomycin and centrifuged at 600 *g* at 4 °C. Aliquots (40 µL) were incubated with an equal volume of 10 mM DNPH at room temperature for 10 min. Successively, 20 µL of sodium hydroxide (6 N) was added, and the absorbance was read at 450 nm. CO content was expressed as µmol CO/mg of protein. The total protein content of the homogenate was determined using the Bradford reagent (Bio-Rad, Milan, IT).

Reactive oxygen species (ROS) were determined by measuring the conversion of 2′,7′-dichlorodihydrofluorescin diacetate (DCFH-DA) to the fluorescent compound dichlorofluorescein (DCF). In brief, homogenate aliquots (25 µg of protein) were mixed with 200 µL of 0.1 M PBS, pH 7.4, containing 10 µM DCFH-DA. FeCl_3_ was added to a final concentration of 100 µM, and the mixture was incubated for 30 min. The conversion of DCFH-DA to the fluorescent product DCF was measured using a microplate reader (Tecan Infinite 200; excitation and emission wavelengths at 485 and 530 nm). The background signal was measured by determining the conversion of DCFH to DCF in the absence of homogenate. A standard curve of DCF was used to calculate the amount of DCF formed, expressed in picomoles. The overall capacity of cells to counteract oxidative stress (ABTS) was determined by measuring the reduction in colour intensity of the 2,2′-azinobis-(3-ethylbenzothiazoline-6-sulfonic acid; ABTS+) radical by cell antioxidants at 734 nm. The ABTS+ radical was formed by incubating the nonradical form of ABTS with potassium persulfate (245 mM) overnight. A calibration curve was generated using a solution of 3,5-Di-tert-4-butylhydroxytoluene (BHT). The total antioxidant capacity was expressed as the equivalent amount of BHT/mg of protein.

### 2.8. Histological Analyses

Nauplii were fixed in Bouin’s solution (15 mL picric acid, 5 mL formalin, 1 mL acetic acid) for 5 min, clarified in xylol and embedded in wax. Serial sections (6 µm) were stained with FITC-conjugated WGA lectin (Vector Laboratories Inc.; Burlingame, CA, USA; 2 mg/mL) to highlight the presence and distribution of d-N-acetyl-glucosamine (glcNAc) in the brush border [40]. Briefly, sections were rinsed in PBS and incubated with the lectin diluted in PBS (0.2 M, pH 7.3; 1 µL in 30 µL buffer) for 15 min at room temperature in a moist chamber in the dark. After rinsing in PBS, binding sites were visualised under a Zeiss Axioskop microscope equipped with UV illumination (excitation at 495 nm, emission at 515 nm). The same observer defined labelling as positive or negative. Negative controls were prepared by incubating slides with the lectins in the presence of the specific competing sugar or by omitting the lectin from the reaction; no autofluorescence was observed.

Fluorescence intensity was quantified by measuring the absorbance on digital photos (400 dpi). Briefly, images of L4 nauplii (n = 25/treatment) were converted to 256-grayscale TIFFs. Using ImageJ (Version 1.54p, 17 February 2025), the densities of n = 50 randomly selected areas were then determined [35].

For TEM, nauplii were fixed in 2.5% glutaraldehyde and 4% paraformaldehyde for 4 h. After repeated washes in 0.1 M PBS (pH 7.4) at 4 °C, samples were post-fixed in 1% osmium tetroxide for 1 h, dehydrated in ethanol and propylene oxide, and embedded in Epon 812 resin (Electron Microscopy Science, Morgantown, PA, USA, MI, IT; 60 °C, 48 h). Serial ultrathin sections (80 nm) obtained from multiple nauplii were collected and analysed. Sections were stained with 3% uranyl acetate and 2.6% lead citrate and observed using a Fei Company, Hillsboro, OR, US; Tecnai G2 transmission electron microscope at 120 kV [41].

### 2.9. Protein Analysis by SDS-PAGE

Nauplii were collected from cultures (n = 3 samples/treatment), pooled, and kept frozen at −80 °C until processing. Samples were homogenised in 50 mM PBS with pH 7.5, containing a protease inhibitor cocktail, sonicated for 2.5 min, and centrifuged at 18,800 *g* for 20 min at 4 °C. The supernatant was collected, and the extracted proteins (40 μg) were run on a 12.5% SDS-PAGE gel in Tris-glycine buffer at 60 mA. Gels were stained with Coomassie blue R-250 [42]. Protein pattern analysis was carried out using ImageJ (version 1.54p, 17 February 2025).

### 2.10. Real-Time PCR

The RNA was extracted from 400 μg of nauplii per treatment using TRI-Reagent (Zymo Research, Irvine, CA, USA) according to the manufacturer’s instructions. RNA concentration and purity were determined by spectrophotometric analysis (NanoDrop, Thermo Fisher Scientific, Wilmington, DE, USA). For each sample, 1 μg of total RNA was reverse-transcribed into complementary DNA (cDNA) using the SuperScript™ VILO™ cDNA Synthesis Kit (Life Technologies, Carlsbad, CA, USA), according to the manufacturer’s protocol. Real-time PCR amplifications were carried out using the Applied Biosystems 7500 Real-Time PCR System and Power SYBR^®^ Green PCR Master Mix (Life Technologies). Reactions were performed in a final volume of 20 μL.

The thermal cycling conditions consisted of an initial denaturation step at 95 °C for 3 min, followed by 40 cycles of denaturation at 95 °C for 15 s and annealing/extension at 60 °C for 1 min. At the end of each run, a melting curve analysis (60–95 °C) was performed to verify amplification specificity. Only reactions showing a single, sharp peak were considered indicative of specific target amplification. Primer specificity and amplification efficiency had been previously validated, and all primer pairs yielded a single product of the expected size, as confirmed by melting curve analysis and agarose gel electrophoresis. Relative gene expression levels were calculated using the comparative Ct (ΔΔCt) method. All experiments were performed in triplicate to ensure reproducibility and minimise technical variability.

### 2.11. Chemicals and Reagents

Unless otherwise specified, all chemicals used in these experiments were AR grade from Sigma Aldrich (Milano, Italy).

### 2.12. Statistical Analyses of Data

Data from quantitative analyses were processed using GraphPad Prism 8 (GraphPad Software, Inc., San Diego, CA, USA). Data normality was assessed using the Shapiro–Wilk test. Statistical differences among groups were evaluated by one-way ANOVA followed by Dunnett’s post hoc test for multiple comparisons versus the control group. A *p*-value < 0.05 was considered statistically significant.

## 3. Results

### 3.1. Cadmium Concentration in Water and Nauplii

No cadmium was detected in seawater (Table 1, line A) and cysts (Table 1, line B). The concentration of the stock solution (nominal Cd conc ~1500 µg/L) was 1015 ± 56.38 µg/L (Table 1, line C) on day 1 and 1256 ± 70.51 µg/L (Table 1, line D) at the end of the experiment, on day 5. In five-day cultures, cadmium remained undetectable in control seawater (Table 1, line E). In cadmium samples, nominal concentrations of 15 and 150 µg/L resulted in measured concentrations of 9.5 and 54.5 µg/L, respectively. Seawater at 1500 µg/L contained cadmium at 1315 ± 176.8 µg/L (Table 1, line H), a value not different from that registered in the stock solution and the 5-day solution without nauplii (Table 1, lines C,D). For simplicity, the nominal values are used consistently in the text and figures.

In nauplii, cadmium showed a moderate but not significant increase in control nauplii (Table 1, line E), probably due to feeding, and a twofold increase in 15 μg/L samples (Table 1, line F). At 150 and 1500 μg/L, nauplii showed a marked increase in cadmium; however, this was not dose-dependent (Table 1, lines G,H).

### 3.2. Effects of Cadmium on Naupliar Oxidative Stress

**Figure 1:**
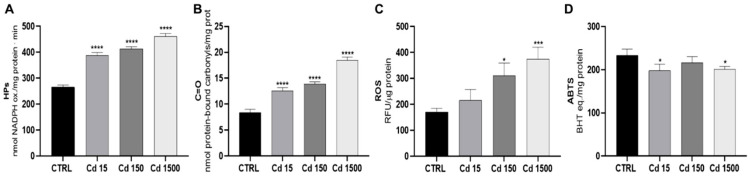
Effects of cadmium contamination on oxidative stress in *Artemia salina* nauplii. Sampling on day 5 post-hatching. Dose-dependent increase in lipid (**A**) and protein (**B**) peroxidation. (**C**) ROS levels increase at the two higher concentrations. (**D**) Reduced total antioxidant capacity at both lower and higher concentrations. Data are means ± SD from six determinations on homogenates derived from pooled nauplii. Difference with respect to controls: *, *p* < 0.05; *** *p* < 0.001; **** *p* < 0.0001.

In nauplii, the hydroperoxides (Figure 1A) and carbonyl groups (Figure 1B) increased dose-dependently, confirming significant lipid and protein peroxidation. Reactive oxygen species (ROS), which did not increase at Cd 15 µg/L, increased dose-dependently at the two higher doses (Figure 1C). The total antioxidant capacity (Figure 1D) significantly decreased at the lower and higher cadmium dosages, remaining unchanged at 150 µg/L.

### 3.3. Effects of Cadmium on Hatching, Mortality and Growth

**Figure 2:**
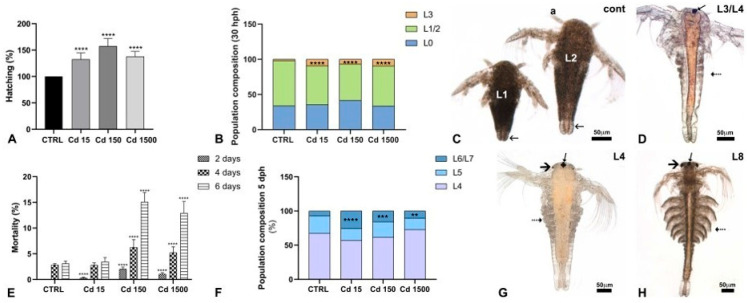
Effects of cadmium contamination on *Artemia salina* development. (**A**) Hatching percentage. (**B**) Composition of the naupliar population at hatching. Note the increased percentage of nauplii at the L3 stage. (**C**,**D**) Stage L1 to L3/L4 nauplii. Antenna II (a), transparent posterior gut (arrows), naupliar eye (arrow), first evidence of trunk segmentation (dotted arrow). (**E**) Increased mortality at the two higher concentrations. (**F**) Changes in composition of the naupliar population on day 5 after hatching. (**G**,**H**) L4 and L8 nauplii. Thoracopods (dotted arrows), naupliar eye (arrows) and paired eyes (thick arrows). Number of animals examined, n > 2000 nauplii/treatment. Difference with respect to controls: **, *p* < 0.01; ***, *p* < 0.001; ****, *p* < 0.0001. Unstained nauplii examined in toto, under incident light. Scale bars: 50 µm.

In controls, the average hatching percentage 30 h after hydration was 16.9 ± 4.5%. This value was set to 100% and used to calculate the hatching percentages in the cadmium samples (Figure 2A).

In the 15 µg/L samples, values increased to 132.3 ± 7.9%, while in the 150 and 1500 µg/L samples, values reached 152.8 ± 11.7% and 139.5 ± 6.2%, respectively. At hatching (Figure 2B), the naupliar population in controls consisted of 31.3 ± 4.8% of nauplii at the L0 stage, still enclosed by the embryonic membranes. The remaining 66.6 ± 1.8% of nauplii were at stages L1/L2 (Figure 2C), with a small percentage (2.1 ± 2.8%) at the L3 stage (Figure 2D). In the samples treated with cadmium (Figure 2B), regardless of the concentration, nauplii at the L3 stage increased significantly, with percentages ranging from 12.4 to 13%. A consequent decrease in L1/L2 nauplii was registered. Notably, in no treatment did the newborn nauplii show signs of distress or death.

As expected, mortality across all samples increased over time (Figure 2E). In controls and in samples exposed to the lower concentration, values remained below 5%. The highest value, 10.5 ± 1.2%, was observed in samples exposed to 150 µg/L cadmium, whereas in those exposed to 1500 µg/L, mortality was 7.6 ± 1.6%.

Population composition was evaluated at the end of exposure (5 dph; Figure 2F). In control samples, 68.2% of nauplii were at stage L4 (Figure 2G), 27.3% were at stage L5, and 4.5% had reached stage L8 (Figure 2H). After exposure to cadmium, the proportion of L6/L7 nauplii notably increased, ranging from a minimum of 10.5% at 1500 µg/L to a maximum of 25.4% at 15 µg/L. Consequently, the proportions of L4 nauplii ranged from 58.7% at 15 µg/L to 73.7% at 1500 µg/L, while those of L5 nauplii varied from 15.8% at 15 µg/L to 21.2% at 150 µg/L.

### 3.4. Teratogenic Effects of Cadmium on Nauplii

**Figure 3:**
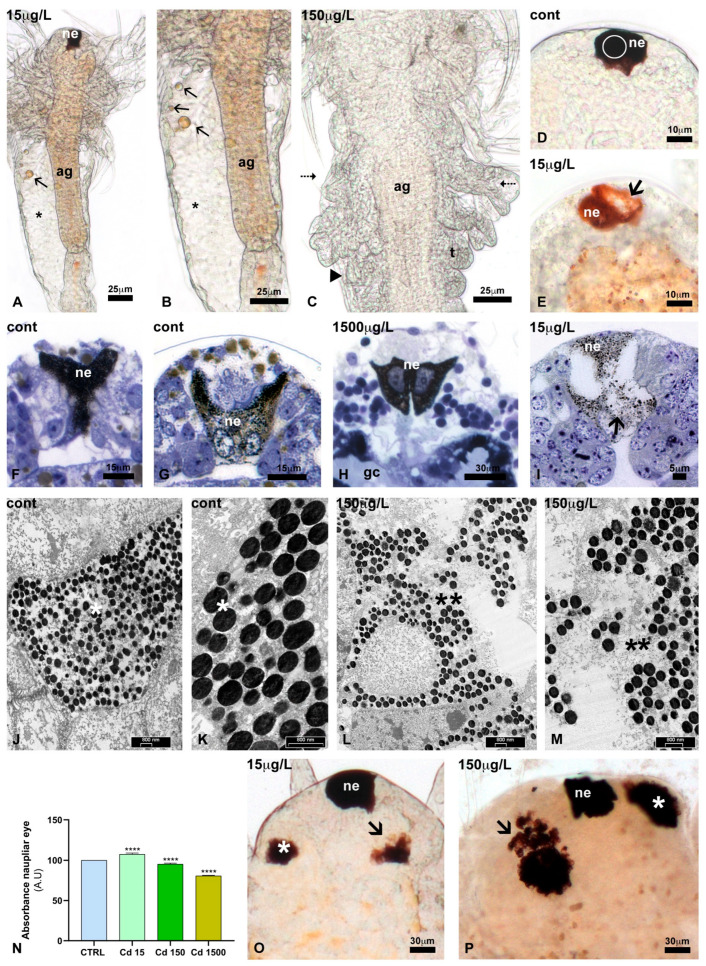
Teratogenic effects of cadmium contamination on *Artemia salina* development. (**A**,**B**) Trunk oedema (*) and presence of large lipid droplets (arrows). Anterior gut (ag), naupliar eye (ne). (**C**) Asymmetric (arrowheads) thoracopods (t). Anterior gut (ag). (**D**) Naupliar eye (ne). White circle: esemplificative area used for determining absorbance. (**E**) Irregularly pigmented (arrow) naupliar eye. (**F**–**H**) Pigmented naupliar eye (ne). Anterior gut (ag), gastric caeca (gc). (**I**) Depigmented (arrow) naupliar eye. Regular (*, **J**,**K**) and irregular (**, **L**,**M**) distribution of pigment granules in the naupliar eye. (**O**,**P**) Representative images of the regular (*) and altered (arrows) paired eyes. Unstained nauplii examined in toto, under incident light (**A**–**E**,**N**,**O**); semithin section stained with toluidine blue (**F**–**I**); ultrathin sections examined at the TEM (**J**–**M**). (**P**) Decrease in pigment density in the naupliar eyes. Measurements (obtained in areas indicated in (**D**)) were obtained using ImageJ (Version 1.54p 17 February 2025). Number of L3/L4 eyes examined = 50/treatment. Difference with respect to controls: **** *p* < 0.0001). Scale bars: 30 µm (**H**,**O**,**P**); 25 µm (**A**–**C**); 15 µm (**F**,**G**); 10 µm (**D**,**E**); 5 µm (**I**); 500 nm (**J**–**M**).

Only modest, dose-independent alterations were observed in body organisation (Table 2, lines A,B). Compared to control nauplii, regardless of the dose, treated nauplii exhibited occasional trunk oedema (Figure 3A,B) or asymmetrical thoracopods (Figure 3C).

More pronounced effects were noted in the eyes. In controls, naupliar eyes had a regular contour (Figure 3D) and were intensely pigmented (Figure 3F,G,J,K). Only occasional alterations were observed (<3% of nauplii; Table 2, line C). However, after Cd exposure, the eyes showed depigmented areas in 16% and 20% of nauplii exposed to 15 and 150 μg/L, respectively (Figure 3E). At the higher concentration, the percentage was lower, at 7.2% (Table 2, line C). Absorbance confirmed a significant decrease in pigment content at the two higher concentrations (Figure 3N).

Paired eyes were absent in early nauplii (Figure 3A,D). First buds appeared at the end of stage L4 (Figure 2G). They were initially asymmetrically pigmented but gradually grew, becoming symmetric and intensely pigmented (Figure 2H). No significant alterations were observed (<5% of nauplii; Table 2, line D). After Cd exposure, significant alterations were observed in 30 to 40% of the nauplii (Table 2, line D). These were unilateral, with no preference for one side over the other. They mainly consisted of reduced pigmentation (Figure 3I,L,M) and/or an irregular disposition of the ommatidia, which determined an irregular eye contour (Figure 3O,P).

A delay in bud development was also observed. At the early L5 stage (Table 2, line E), 89.9% of control nauplii possessed two clearly visible paired buds. In contrast, in Cd-exposed nauplii, the percentage decreased dose-dependently, with only 36.5% exhibiting regularly developed paired eye buds at the highest Cd concentration (96.7% and 100%, respectively; Table 2, line E). By the early L6 stage (Table 2, line F), well-developed paired buds were present in 100% of control nauplii and in nauplii exposed to the two highest Cd concentrations. At the lowest concentration, the percentage remained at 89.7. By stage L7, all nauplii had regular paired eyes.

### 3.5. Effects of Cadmium on Lipid Reserve

**Figure 4:**
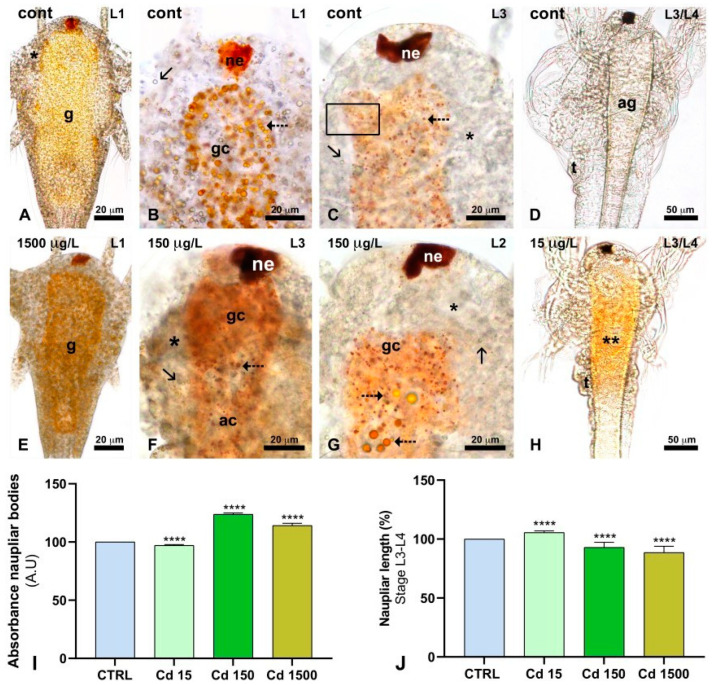
Effects of cadmium on the presence and distribution of embryonic reserves in *Artemia salina* nauplii. (**A**) Yolk globules (yellow) in the gut (g) and lipid droplets in the body wall (*). (**B**) Detail. Lipid droplets (arrow) and yolk globules (dotted arrow). Gastric caeca (gc), naupliar eye (ne). (**C**) Small residual lipid droplets (arrow) and yolk globules in the gut (dotted arrow). Black box: esemplificative area used for determining absorbance. (**D**) Transparent body wall and anterior gut (ag). Thoracopods (t). (**E**,**F**) Dense body wall (*), lipid droplets (arrows), and yolk platelets (dotted arrows). (**G**) Dense body wall (*) due to large residual yolk globules in the gut (dotted arrows) and lipid droplets (arrow). (**H**) Residual yolk in the anterior gut (**). Thoracopods (t). Unstained nauplii examined in toto, under incident light. (**I**) Quantitative analysis of body density. Measurements, obtained in areas indicated in (**C**). (**J**) Naupliar length at the L3/L4 stage. Graph data were obtained using ImageJ (Version 1.54p, 17 February 2025) (n = 75 nauplii/treatment; difference with respect to controls: **** *p* < 0.0001). Scale bars: 50 µm (**D**,**H**); 20 µm (**A**–**C**, **E**–**G**).

Stage L1 nauplii, both controls (Figure 4A,B) and those exposed to cadmium at different concentrations (Figure 4E), displayed short, dense bodies due to yellow yolk platelets and transparent lipid vesicles. In control nauplii, during stages L2 to L3, reserves were progressively resorbed (Figure 4C), so that by stage L4 the body was transparent (Figure 4D). In nauplii exposed to cadmium, reserve resorption was delayed; as a result, L3 nauplii still exhibited a dense body (Figure 4F). Occasionally, large yolk platelets appeared, no matter the naupliar stage, especially at the two highest concentrations (Figure 4G). At stage L3/L4, nauplii retained significant reserves, so their bodies appeared somewhat denser, with the anterior gut distinctly yellowish, indicating the significant presence of yolk (Figure 3H). Absorbance confirmed the increased density of the naupliar bodies at stage L3/L4 after exposure to the two higher Cd concentrations (Figure 4I). In the same nauplii, length was increased at 15 µg/L and significantly decreased at the two higher concentrations (Figure 4J).

### 3.6. Effects of Cadmium on Gut Epithelium

**Figure 5:**
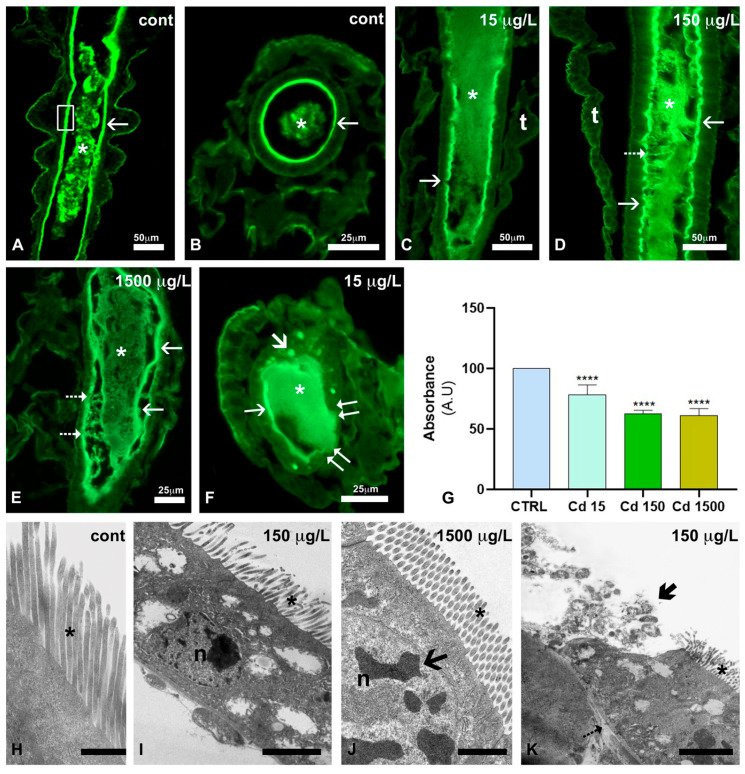
Effects of cadmium on the enterocyte brush border in *Artemia salina* nauplii exposed to 15, 150 and 1500 µg/L cadmium. (**A**–**F**) Presence and distribution of glcNAc. (**A**,**B**) Intensely labelled brush border (arrows) and food remnants (*) in the lumen. White box: esemplificative area used for determining absorbance. (**C**) Moderately discontinuous brush border (arrow). Note the diffuse labelling of the gut lumen (*). Thoracopods (t). (**D**,**E**) Discontinuous and irregular brush border (arrows). Note the fluorescent filamentous material apparently detaching from the epithelial surface (dotted arrows). Food remnants (*) in the lumen. (**F**) Dense bodies in enterocytes (arrow). Labelled brush border (thin arrow) and luminal content (*). Note the tract of eroded epithelium (double arrows). Sections stained with the lectin WGA conjugated with FITC, observed under UV light. (**G**) Quantitative analysis of fluorescence. Measurements were obtained in areas indicated in Fig. (**A**) using ImageJ (Version 1.54p, 17 February 2025) (n = 50/treatment. Difference with respect to controls: **** *p* < 0.0001). (**H**) Regular organisation of the brush border microvilli (*). (**I**) Moderately altered brush border (*). Enterocyte nucleus (n). (**J**) Regular brush border (*). Nucleus (n) with patchy chromatin (arrow). (**K**) Moderately altered brush border (*). Note the eroded epithelium with residues (thick arrow) released in the lumen (l). Basal lamina (dotted arrow). Scale bars: 50 µm (**A**,**C**,**D**); 25 µm (**B**,**E**,**F**); 2.5 µm (**K**); 2 µm (**I**); 1.5 µm (**J**); 750 nm (**H**).

In controls, WGA stained the gut brush border intensely; no differences were observed across developmental stages (Figure 5A,B). In the gut lumen, staining was also observed on algal food remnants. In nauplii exposed to Cd, the brush border appeared discontinuous and irregularly outlined (Figure 5C–E). Gut content appeared dense and diffuse at 15 µg/L (Figure 5C,F), whereas at 150 µg/L (Figure 5D) and 1500 µg/L (Figure 5E), the lumen was invaded by filamentous material, apparently detaching from the epithelium. Occasional dense bodies were observed in the enterocytes (Figure 5F). Absorbance confirmed a dose-dependent decrease in the presence of N-Ac galactosamine in the brush border (Figure 5G).

At the TEM, control epithelial cells exhibited a high, well-organised brush border with long, regularly oriented microvilli (Figure 5H). After exposure to 15 μg/L Cd, no significant differences were observed. In contrast, at the two higher concentrations, large patches of tissue showed microvilli that exhibited low to moderate disorganisation (Figure 5I,K). Ulcerations were also observed, with consequent release of cell debris into the lumen (Figure 5F,K).

### 3.7. Effects of Cadmium on Protein Pattern and hsp26 and hsp60 Expression

**Figure 6:**
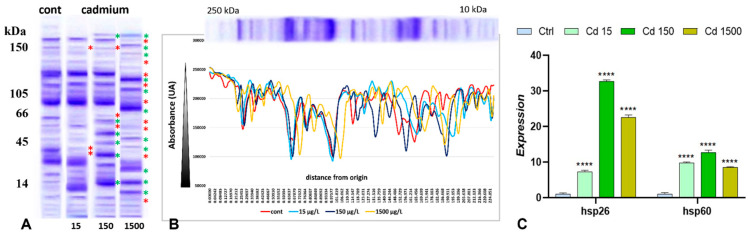
Effects of cadmium on protein pattern of 5-day post-hatching nauplii of *Artemia salina*. (**A**) SDS gel stained with Coomassie blue. The main differences relative to the control are indicated by asterisks (red for bands disappearing, green for new bands). (**B**) Densitometric analysis of the gel shown in Figure (**A**). Absorbance increases with band density (grey value for black is 0, and for white is 255). On the left: origin of the gel (loading wells). The data plotted were obtained with ImageJ (Version 1.54p 17 February 2025; accessed on 6 November 2025). (**C**) Expression of *hsp26* and *hsp60*. Note the absence of a clear dose-dependency (n = 3 samples/treatment; difference with respect to controls: **** *p* < 0.0001).

The SDS-PAGE analyses demonstrated that in 5-day control nauplii, the protein pattern showed several bands ranging from 200 to 10 kDa (Figure 6A). After exposure to cadmium, significant changes were observed, with several bands either disappearing, appearing, or changing in intensity (Figure 6A). Densitometric analyses conducted with ImageJ (v1.54p) confirmed the observed variations in band presence and intensity (Figure 6B).

The expression of the two *hsp* genes increased significantly after Cd exposure, but no clear dose-dependence was observed. Higher values, in fact, were registered at 150 μg/L Cd (Figure 6C). In addition, *hsp26* was more significantly over-expressed with respect to *hsp 60*.

## 4. Discussion

Water analysis showed that the nominal Cd concentration of about 1500 µg/L, calculated from the CdCl_2_ formula, corresponded to a real concentration of about 1100 µg/L. Lower values are explained by assuming that part of the Cd adhered to the glass culture tank walls [43,44], to the naupliar surface, or was retained in the faeces (not collected for water analysis).

In accordance with [31], nauplii exposed to 15 µg/L did not accumulate relevant amounts of Cd, as indicated by a real value lower than 0.1 µg/gr. In contrast, at 150 µg/L, a concentration that is not environmentally compatible, Cd began to accumulate, but no differences were observed relative to the concentration measured at 1500 µg/L. In both cases, values remained at about 9 µg/gr. This finding, although seemingly contradictory, aligns well with the literature, indicating that in *Artemia salina*, uptake is inversely related to Cd concentrations, whereas elimination is directly related to Cd concentrations [31]. Higher exposures, therefore, do not necessarily correspond to higher uptake [36]. This evidence demonstrates that Cd binding sites can be saturated [45,46] and, therefore, that toxicokinetics do not depend only on the activation of efficient detoxifying mechanisms [47]. Their activation is confirmed by the absence of significant changes in total antioxidant capacity. *Artemia* nauplii, therefore, are very resistant to the metal, at least for short exposure periods, evidence confirmed by the absence of mortality. A prompt defensive response is consistent with an evolutionary adaptation in larvae to changing metal levels in unstable aquatic environments [48].

Mortality rates demonstrate the effectiveness of the activated protective mechanisms. Although at the two higher Cd concentrations the percentage reached 15%, the value remained close to that accepted for control cultures [37]. Therefore, the high tolerance of *Artemia* is confirmed [49]. In *Artemia franciscana*, the LC50 is 3 mg/L [50], whereas in *Artemia parthenogenetica*, the LC50 ranges from 0.5 to 17 mg/L [28].

Increased oxidative stress and mortality do not align with the results obtained for hatching rate and growth. In fact, for both parameters, a positive, stimulatory effect of Cd was registered. At the lower dose, a possible hormetic effect can be observed [51,52]. The modest metal uptake stimulated metabolism and embryo development, favouring naupliar growth. On the contrary, it is more difficult to explain how this was possible at the two higher concentrations, which were associated with higher oxidative stress and Cd concentration in tissues of about 8 µg/g. One explanation is that the Cd absorbed by the nauplii was below the toxicity threshold in both cases [53]. In adult *Artemia,* the LC10 is 58 mg/L [28], while in nauplii, the estimated LC50 values vary with population, settling around 120–150 mg/L [28]. If this is the case, the nauplii cope with increased Cd levels in their tissues by activating antioxidant responses, which may include upregulation of metallothionein expression [54]. Support for this hypothesis comes from the altered protein patterns and the increased expression of *hsp26* and *hsp60*, indicating an effort to enhance metal tolerance [55] and maintain cellular homeostasis [56]. Another possibility is that Cd activated the Nrf2-mediated oxidative stress response pathway, and the dynamic interplay between ubiquitination/deubiquitination [57]. This activation could have favoured ROS scavenging, as well as cell cycle progression and, consequently, development [58]. Further investigation aiming at characterising the observed changes in protein expression will help clarify these adaptive responses.

As far as Cd teratogenicity is concerned, alterations were already present at 0.5 µg/g, in agreement with the observed lipid and protein peroxidation. Surprisingly, in most cases, they were more significant than those exerted at 8 µg/g. This discrepancy may be due to the fact that uptake was measured in total homogenates. Cd accumulates at different rates in different tissues [59,60] and, therefore, at the moment, we cannot correlate concentrations with specific effects on specific targets. In addition, *Artemia* nauplii are a rapidly developing system in which toxic damage depends not only on concentration but also on the target and the extent of interference exerted on transcriptomics [61].

Toracopods and eyes were the most affected. Significantly, the same effects were observed after exposure to benzodiazepines, which also caused significant oxidative stress [35]. In thoracopods, the alterations consisted of asymmetrical development of the first pair, its earlier development, and better organisation at the time of sampling. The thoracopod anomaly suggests variations in gene expression, particularly in mechanisms responsible for division and differentiation [62] of ventral epidermal cells [63]. It is easy to postulate that such interference was induced by oxidative stress.

Whatever the mechanism activated by Cd, the observed damage suggests that motor performance should be verified, as effects of Cd on swimming have already been reported in *Branchipodopsis* [64] and in *Danio rerio* [65]. The metal, in fact, interferes with skeletal muscle organisation by impairing homeostasis [66] and dysregulating several genes [67], with structural and functional consequences [68].

The other evident damage was to the naupliar eyes, where marked depigmentation was observed. Metals have been reported to reduce the number of chromatophores and cause melanin degeneration [69]. Consequently, it cannot be excluded that light perception could be altered, as already demonstrated in *Danio rerio* [70]. Nauplii are planktonic larvae that move vertically in the water column in response to day/night illumination cycles. Therefore, inefficient eyes would impair such migration and have severe consequences for feeding and escape from predators.

Reduced pigmentation indicates that Cd downregulated the expression of genes implicated in melanisation [71], particularly microphthalmia-associated transcription factor (MITF) and tyrosinase, two genes that are under the sequential control of cAMP/protein kinase A (PKA/CREB), mitogen-activated protein kinase (MAPK) and Wnt signalling [72]. It is intriguing that, in spiders, MITF also controls appendage regeneration, and that, in the presence of Cd, partial depigmentation and anatomical defects were affected [71]. The findings of depigmented eyes and altered thoracopods in *Artemia salina* appear, therefore, to be justified by a single altered developmental pathway.

Depigmentation may also explain the delay observed in the appearance of paired eye buds. In summary, early buds were present but composed of only a few poorly depigmented elements, and thus invisible to in toto observation. By stage L6/L7, the ommatidia became more numerous, and consequently, the eyes became visible. Ongoing transmission electron microscopy (TEM) investigations will provide details on pigment appearance and distribution, and, possibly, on ommatidia disposition. In fact, in toto, observations also indicated frequent disorganisation. In *Artemia salina*, paired-eye formation is a complex process that requires the proliferation of neuronal stem cells and the expression of synapsin-like proteins [73]. Cd interference with stem neural cell proliferation is a well-known effect [74], mediated by p53, PI3K-AKT, MAPK, and NF-κB signalling pathways [75].

Of particular interest is the finding that cadmium delayed the resorption of embryonic reserves and caused the formation of large yolk and lipid droplets. A parallel decrease in naupliar length was observed, as expected, considering that *Artemia salina* is a typical lecithotrophic species. The embryos and early nauplii rely on abundant reserves to sustain early development [34], both for synthesising new tissues and for providing the necessary energy. At hatching, the transparent lipid droplets and yellow yolk platelets are dispersed throughout the body. Then, they gradually reduce in size, disappearing from the body wall, the hindgut and, by the L3/L4 stage, also from the foregut [76]. Progressive consumption triggers exogenous feeding during stage L3.

The evidence that Cd interfered with lipid resorption aligns well with the metal’s ability to dysregulate genes and enzymes involved in lipid metabolism [77]. Oxidative stress may be the leading cause, via peroxidation and mitochondrial dysfunction [78]. The same goes for yolk. Peroxidation may have altered its lipo- and protein components, the enzymes involved in degradation (cathepsin L/B) [79] or their expression [80].

Interestingly, in Cd-exposed nauplii, yolk globules persisted longer in the anterior gut, the site at which most of the Cd is uptaken [36]. A possible explanation is that the anterior enterocytes, exposed to greater levels, were damaged. In crustaceans, yolk is degraded within the embryo’s cells, specifically in specialised organelles called yolk granules, which function like lysosomes [81]. The analysis of the brush border confirms our hypothesis, clearly demonstrating structural and molecular alterations at this level. Preliminary TEM analyses also evidence areas of epithelial erosion, further confirming metal toxicity to enterocytes, including possible interference with cell-to-cell adhesion [82]. Erosion explains the significant change observed in the gut lumen content of the treated animals.

Electrophoretic analyses may reveal Cd-induced changes in protein expression patterns, suggesting potential interference with developmental processes. So far, no band has been identified. However, based on the literature, changes are expected in antioxidant enzymes and Cd-chelating metallothioneins [47]. Caspases should also be upregulated to eliminate damaged cells [83,84], while transcriptomic and metabolomic changes adapt the nauplius to altered metabolic pathways [85,86].

## 5. Conclusions

In conclusion, Cd affects the development of *Artemia salina* nauplii by inducing oxidative stress and altered protein expression. The most evident consequence is a delay in yolk resorption, resulting in interference with the larval metabolic pathways and reduced growth. Minor alterations observed in thoracopod and paired-eye development would probably interfere with naupliar fitness and survival in a natural, competitive environment.

The mechanisms of action need further clarification, as several aspects remain unclear. Gaining a better understanding is crucial because crustacean larvae are a vital part of zooplankton, and any interference could lead to serious environmental impacts across the entire food chain.

## Figures and Tables

**Table 1:** Cd concentration in control seawater (A) and in cysts (B). Measured Cd concentration in the stock solution (nominal conc 1500 µg/L Cd2+) at preparation (C) and after 5 days (D). (E–H) Left column, treatments; middle column: measured Cd concentrations in seawater; right column: measured Cd concentrations in nauplii. Nominal concentration (nom); control (cont); 5 days post-hatching (5 dph). Difference with respect to controls: * *p* < 0.05; **** *p* < 0.0001. Values are means ± SD; n = 4 replicates/treatment. Estimated detection limit (LOD) is 1–5 ng/gr.

		Water	Cysts/Nauplii
A	artificial seawater	<1 µg/L	
B	cysts		<0.01 µg/gr
C	Cd 1500 µg/L (nom) T0	1015 ± 56.38 µg/L	
D	Cd 1500 µg/L (nom), T5 dph	1265 ± 70.51 µg/L	
E	cont, 5 dph	<1 µg/L	<0.05 µg/gr
F	15 µg/L (nom), 5 dph	9.5 ± 2.1 µg/L	<0.1 µg/gr *
G	150 µg/L (nom), 5 dph	54.5 ± 9.2 µg/L	7.86 ± 0.8 µg/gr ****
H	1500 µg/L (nom), 5 dph	1315 ± 176.8 µg/L	9.93 ± 1.1 µg/gr ****

**Table 2:** Incidence of morphological alterations in *Artemia salina* nauplii exposed to cadmium. Values, reported as percentages, are means ± SD. Difference with respect to controls: * *p* < 0.05; ** *p* < 0.01; *** *p* < 0.001. Nauplii examined, n ≥ 2000/treatment; replicate experiments, n = 4. Control (cont); cadmium (Cd) nominal concentrations.

		Cont	15 μg/L Cd	150 μg/L Cd	1500 μg/L Cd
A	trunk oedema (stage L2/L3)	0.5%	2.2%	2.1%	1.4%
B	thoracopod asymmetry (stage L4/L5)	≤1%	1.8%	1.2%	1.6%
C	naupliar eye depigmentation (stage L2/L3)	<3%	16 ± 2.9% ***	20.3 ± 1.3% ***	7.2 ± 4.5% **
D	dysmorphic paired eye buds (stage L5/L6)	4.9 ± 1.6%	33.4 ± 9.1% **	40 ± 12.6% ***	29.6 ± 7.2% *
E	two eye buds (stage L4/L5)	89.9 ± 11.3%	72.8 ± 9.7% *	55.8 ± 17.6% **	36.5 ± 19.4% ***
F	two eye buds (stage L5/L6)	100%	89.7 ± 3.4% *	96.7 ± 1.5%	100%

## Data Availability

The original contributions presented in this study are included in the article. Further inquiries can be directed to the corresponding authors.
